# Brain Gray Matter Atrophy and Functional Connectivity Remodeling in Patients With Chronic LHON

**DOI:** 10.3389/fnins.2022.885770

**Published:** 2022-05-12

**Authors:** Qin Tian, Ling Wang, Yu Zhang, Ke Fan, Meng Liang, Dapeng Shi, Wen Qin, Hao Ding

**Affiliations:** ^1^Department of Medical Imaging, People's Hospital of Zhengzhou University, Henan Provincial People's Hospital, Zhengzhou, China; ^2^Department of Radiology, Tianjin Medical University, Tianjin, China; ^3^Tianjin Key Laboratory of Functional Imaging, Tianjin Medical University General Hospital, Tianjin, China; ^4^Henan Eye Institute, Henan Eye Hospital, Henan Provincial People's Hospital, Zhengzhou University People's Hospital, Zhengzhou, China; ^5^School of Medical Imaging, Tianjin Medical University, Tianjin, China

**Keywords:** LHON, gray matter volume (GMV), functional connectivity (FC), visual cortex (V1), reorganization

## Abstract

**Purpose:**

The aim of this study was to investigate the brain gray matter volume (GMV) and spontaneous functional connectivity (FC) changes in patients with chronic Leber's hereditary optic neuropathy (LHON), and their relations with clinical measures.

**Methods:**

A total of 32 patients with chronic LHON and matched sighted healthy controls (HC) underwent neuro-ophthalmologic examinations and multimodel magnetic resonance imaging (MRI) scans. Voxel-based morphometry (VBM) was used to detect the GMV differences between the LHON and HC. Furthermore, resting-state FC analysis using the VBM-identified clusters as seeds was carried out to detect potential functional reorganization in the LHON. Finally, the associations between the neuroimaging and clinical measures were performed.

**Results:**

The average peripapillary retinal nerve fiber layer (RNFL) thickness of the chronic LHON was significantly thinner (T = −16.421, *p* < 0.001), and the mean defect of the visual field was significantly higher (T = 11.28, *p* < 0.001) than the HC. VBM analysis demonstrated a significantly lower GMV of bilateral calcarine gyri (CGs) in the LHON than in the HC (*p* < 0.05). Moreover, in comparison with the HC, the LHON had significantly lower FC between the centroid of the identified left CG and ipsilateral superior occipital gyrus (SOG) and higher FC between this cluster and the ipsilateral posterior cingulate gyrus (*p* < 0.05, corrected). Finally, the GMV of the left CG was negatively correlated with the LHON duration (*r* = −0.535, *p* = 0.002), and the FC between the left CG and the ipsilateral posterior cingulate gyrus of the LHON was negatively correlated with the average peripapillary RNFL thickness (*r* = −0.522, *p* = 0.003).

**Conclusion:**

The atrophied primary visual cortex of the chronic LHON may be caused by transneuronal degeneration following the retinal damage. Moreover, our findings suggest that the functional organization of the atrophied primary visual cortex has been reshaped in the chronic LHON.

## Introduction

Leber's hereditary optic neuropathy (LHON) is a maternally inherited disease, 90% of which is caused by three primary mutation sites, namely, 11778/ND4, 3460/ND1, and 14484/ND6, affecting different subunits of complex I and leading to dysfunction in the mitochondrial respiratory chain (Carelli et al., 2004; Yu-Wai-Man et al., 2009; Giordano et al., 2011). Clinically, the LHON is marked by usually sequential subacute vision loss with bilateral central scotomas that occur most frequently in young men (Ollinger et al., 2001). The retinal ganglia cells (RGCs) and small-caliber fibers of the papillomacular bundle are selectively lost in the early stages of the pathologic process. As the disease progresses, the peripapillary retinal nerve fiber layer (RNFL) becomes thinner and optic nerve atrophy happens (Shulman et al., 2009).

Brain white matter (WM) and gray matter (GM) abnormalities were consistently reported in the chronic LHON, especially along the brain's visual pathways. For example, Barcella et al. reported that the optical radiation was atrophied in the chronic LHON using voxel-based morphometry (VBM) (Barcella et al., 2010). Microstructural changes in the optical radiation in the chronic LHON, as represented by decreased fractional anisotropy (FA) and increased mean diffusivity (MD) and radial diffusivity (RD), were also confirmed by several recent studies (Milesi et al., 2012; Rizzo et al., 2012; Ogawa et al., 2014; Manners et al., 2015; Takemura et al., 2019). Besides the WM changes, VBM also detected decreased GM volume (GMV) in the primary visual cortex (Barcella et al., 2010). Postmortem examination showed a significant neuron loss in both the parvocellular and magnocellular layers of the lateral geniculate nucleus (LGN). These pieces of evidence support the secondary transneuronal degeneration of the visual pathway driven by the primary involvement of the RGCs in the LHON.

Despite the secondary damages, compensatory changes in the brain were also reported in the patients with LHON or carriers. The V2 and V3, two visual association areas, were thicker in the young asymptotic LHON carriers than in the healthy controls (HCs), indicating a potential plastic change in these regions following early retinal involvement (d'Almeida et al., 2013; Mateus et al., 2016). Furthermore, the synchronization of spontaneous blood oxygen level-dependent (BOLD) fluctuations was higher in the high-level visual and auditory networks, which was accompanied by reorganized topology and structural connectivity of the auditory subareas (Rocca et al., 2011). Thus, the damage and compensatory plasticity may coexist in the brain of the LHON. However, it is unknown that the degenerated visual areas also had functional plastic potentials in the chronic LHON. In this study, we hypothesized that the atrophy of the visual cortex in the chronic LHON will lead to functional connectivity (FC) reorganization in the remote brain areas. This hypothesis is based on early studies about the functional impairment and remodeling after blindness by ocular diseases or injuries (Qin et al., 2015). For example, an early study reported that the FC between frontoparietal network and visual cortex was enhanced in the patients with congenital blindness (Qin et al., 2015).

To test this hypothesis, a relatively large sample of patients with chronic LHON and matched sighted control group were recruited to identify the visual areas with decreased GMV in the chronic LHON, to detect the changes in resting-state FC between the atrophied brain areas and the whole brain, and to explore the relationship between structural/functional changes of the brain and clinical measures.

## Materials and Methods

### Participants

A total of 32 patients with chronic LHON (12 women and 20 men, age range: 27.97 ± 11.85 years) and 32 matched sighted HCs (10 women and 22 men, age range: 25.56 ± 9.55 years) were also enrolled from Henan Provincial People's Hospital. The inclusion and exclusion criteria for the LHON cohort were: (1) all patients are genetically confirmed with one of the three primary point mutations of mtDNA (m.3460G > A, m.11778G > A, and m.14484T > C); (2) have a duration after vision loss larger than 1 year; (3) have no history of neurological/psychiatric diseases or substance abuse; (4) have no brain and spinal abnormalities using routine magnetic resonance imaging (MRI); and (5) have no other ophthalmic diseases, such as glaucoma, cataract, and retinopathy. The HCs were enrolled with the same criteria except for the diagnosis of LHON. The detailed demographic information of the recruited subjects is provided in Table 1. This study was approved by the local ethics committee, and written consent was obtained from the participants or their guardians for immaturity. The peripapillary RNFL thickness and mean defect of the visual field (MD_VF_) were measured at enrollment. The average RNFL (360° measure) thicknesses were quantified using a high-resolution spectral-domain Cirrus platform (Carl Zeiss Meditec, Dublin, CA, USA).

**Table 1:** Demographic information of the recruited subjects.

	**Chronic LHON**	**HC**	**Statistics**	***P* value**
Gender [males /females]	20/11	22/9	χ^2^ = 0.93	0.393
Age (range) [years]	27.65 ± 11.93 (13–53)	25.16 ± 9.43 (11–44)	t = −0.91	0.177
LHON duration [months]	119.6 ± 129.2	-	-	-
Peripapillary RNFL thickness [μm]	59.62 ±10.93	100.60 ± 6.98	t = −16.42	<0.001
MD_VF_[%]	17.99 ± 7.91	1.57 ± 1.02	t = 11.28	<0.001

*LHON, Leber hereditary optic neuropathy; HC, healthy controls; MD_VF_, mean defect of visual field; RNFL, retinal nerve fiber layer*.

### MRI Acquisition

The MRI data were obtained on a GE Discovery MR750 3.0T MR scanner (GE Healthcare, Waukesha, WI, USA) with an 8-channel head receiver coil. The routine MRI was initially performed to exclude subjects with brain and spinal abnormalities. High-resolution 3D T1-weighted images were acquired using a brain volume (BRAVO) sequence with parameters as follows: repetition time (TR) = 8.2 ms, echo time (TE) = 3.2 ms, inversion time (TI) = 450 ms, matrix size = 256 × 256, field of view (FOV) = 256 mm × 256 mm, flip angle = 12°, slice thickness = 1 mm, and 176 slices with no gap. The resting-state functional MRI (fMRI) images were obtained using a single-shot gradient-echo echo-planar imaging sequence: TR = 2,000 ms, TE = 30 ms, flip angle = 90 degree, matrix = 64 × 64, FOV = 22 × 22 cm, slice thickness =3.4 mm, gap = 1.0 mm, 33 slices, interleaved transverse slices, and 210 volumes. All subjects were asked to keep awake with eyes closed and heads static during the fMRI scan. One patient with chronic LHON and one sighted control were excluded from the imaging analysis due to poor MRI data quality.

### Structural MRI Preprocessing

All 3D T1-weighted structural data were preprocessed using the VBM8 toolbox implemented in the Statistical Parametric Mapping (SPM) software version 12 (http://www.fil.ion.ucl.ac.uk/spm). During the segmentation, an adaptive maximum *a posterior* technique and a partial volume estimation were applied to estimate the fraction of each pure tissue type present in every voxel. Following the segmentation of GM, WM, and cerebrospinal fluid (CSF), the individual GM and WM components were normalized into the Montreal Neurological Institute (MNI) space using the diffeomorphic anatomical registration through the exponentiated Lie algebra (DARTEL) algorithm based on a study-specific DARTEL template, and re-sliced to a voxel size of 1.5 × 1.5 × 1.5 mm^3^. The study-specific DARTEL template was created using the structural data of all recruited subjects based on the standard pipeline provided by SPM12. Then, the relative GMV or WM volume (WMV) maps of each subject were obtained by multiplying the individual's GM or WM component by the non-linear Jacobian determinants derived from the DARTEL deformation parameters to remove the confounding effects of individual global brain volume. Finally, the GMV and WMV maps were smoothed with a full width at half maximum (FWHM) kernel of isotropic 6 mm. After the spatial preprocessing, the normalized and smoothed GMV and WMV maps were used for the following analysis.

### Resting-State FMRI Preprocessing

The resting-state fMRI data were preprocessed using the DPARSFA toolbox version 4.3 (rfmri.org/dpabi) and SPM with the following steps: the first 10 volumes of each subject were discarded for removing the effect on the potential fMRI signal dropping caused by incomplete T1 relaxation. The remaining 200 volumes underwent slice-timing to correct the acquisition time delay between slices. Anda rigid spatial realignment was performed to estimate and correct for head motion-induced displacement among scan volumes. All resting-state fMRI data were under the head-motion thresholds with a maximum translation of <2 mm and a maximum rotation of <2.0° in any direction. Considering signal spikes caused by head motion can significantly contaminate the final resting-state fMRI quantification, the frame-wise displacement (FD) was also calculated based on the head motion parameters, representing the volume-by-volume head motion amplitude. The realigned fMRI images of each subject were then linearly co-registered with the individual structural images and were further transformed into the MNI space using the DARTEL deformation field generated during T1 normalization as mentioned above, which were further resampled into a voxel size of 3 × 3 × 3 mm^3^. The Friston-24 head motion parameters (six motion parameters and their first-time derivatives, and such 12-corresponding squared items), the volumes with motion spike (FD > 0.5), and the average BOLD signals of the ventricular, WM, and global brain tissue were regressed out from the fMRI data. The regressed fMRI data were subsequently band-pass filtered with a frequency range of 0.01–0.08 Hz to remove high-frequency noises and low-frequency signal drifts. Finally, the filtered fMRI data were smoothed with an FWHM kernel of 6 mm for later quantification.

### Voxel-Wise Statistics of the Gray and White Matter Volume

The non-parametric permutation tests (5,000 shuffles) were, respectively, performed to investigate voxel-wise GMV and WMV differences between the chronic LHON and HC groups while controlling for the effects of age and gender using the “randomize” script of FSL 6.0 (https://fsl.fmrib.ox.ac.uk/fsl/fslwiki/). Multiple comparisons were corrected using a threshold-free cluster enhancement (TFCE) family-wise error (FWE) method (*p* < 0.05).

### Voxel-Wise Functional Connectivity Calculation and Statistics

The brain regions with intergroup differences in GMV from the VBM step were extracted as the seeds for FC calculation. Specifically, voxels survived during the VBM (*P* < 0.05, FWE corrected) and were within a 9-mm radius sphere that centered at the statistic peak (left calcarine gyrus [CG]: [−10.5, −66, −3]; right CG: [15, −78, 1.5]) were defined as the FC seeds. Regions of interest (ROIs) are a method of marking specific parts of an image. The common approach for investigative ROIs analysis is to create small ROIs at the peak of a threshold cluster (Poldrack, 2007). The spheres are defined by the small radius to ensure that the spheres only include voxels that are more significant. Consistent with the previous studies (Niedtfeld et al., 2010; Li et al., 2017; Zhou et al., 2019), we defined the ROI by a sphere of 9 mm (3 voxels) of radius (about 33 voxels in this sphere) at the peak of group-wise difference clusters based on an isotropic spatial resolution of 3 × 3 × 3 mm^3^ for the resting-state fMRI. The mean time series of BOLD signals of the bilateral CGs were then extracted, and the Pearson correlation coefficients between the time series of the seed ROIs and that of each voxel of the whole-brain GM were computed. Later, a Fisher r-to-z transformation algorithm was introduced to convert the correlation coefficient into z-value to increase normality.

Voxel-wise non-parametric permutation tests (5,000 shuffles) were performed to investigate FC differences between the chronic LHON and HC groups while controlling for the effects of age and gender (*p* < 0.05, TFCE-FWE corrected).

### Other Statistics

The Kolmogorov–Smirnov tests were first introduced to assess the normality of the variables, including the age, duration, neuro-ophthalmologic assessments, and ROI-wise MRI metrics. The type of ROI-wise MRI metrics was referred to group-wise differences in GMV and FC, including GMV of bilateral CG, and resting-state FC between the left superior occipital gyrus (SOG) and posterior cingulate gyrus. All variables except for the duration were normally distributed. Thus, paired *t*-test was used to test the statistical differences in neuro-ophthalmologic measures between the left and right eyes in the LHON (*p* < 0.05). A two-sample Student's *t*-test was used to test the intergroup differences in neuro-ophthalmologic measures and age (*p* < 0.05). A chi-squared test was used to test the intergroup differences in gender (*p* < 0.05). Finally, the Spearman (non-normality) or Pearson correlation (normality) was used to test the associations between clinical measures and MRI metrics (*p* < 0.05, Bonferroni correction). These statistics were carried out using the SPSS version 19 (https://www.ibm.com/analytics/spss-statistics-software).

## Results

### Demographic and Clinical Measurements

As shown in Table 1, there were no statistical differences in either age (Student's *t*-test, t = −0.910, *p* = 0.177) or genders (chi-squared test, χ^2^ = 0.925, *p* = 0.393) between the chronic LHON and HC. For the LHON, the duration of visual loss ranged from 13 to 409 months for both eyes. The paired *t*-test showed no statistical differences in mean peripapillary RNFL thickness (t = 0.824, *p* = 0.417) and MD_VF_ (t = 1.662, *p* = 0.107) between the left and right eyes. Thus, these clinical measures of bilateral eyes were merged for further statistics. Compared with the HC, the Student's *t*-test identified significantly thinner peripapillary RNFL (t = −16.42, *p* < 0.001) and higher MD_VF_ (t = 11.28, *p* < 0.001) in the chronic LHON.

### Intergroup Differences in Neuroimaging Measurements

**Figure 1:**
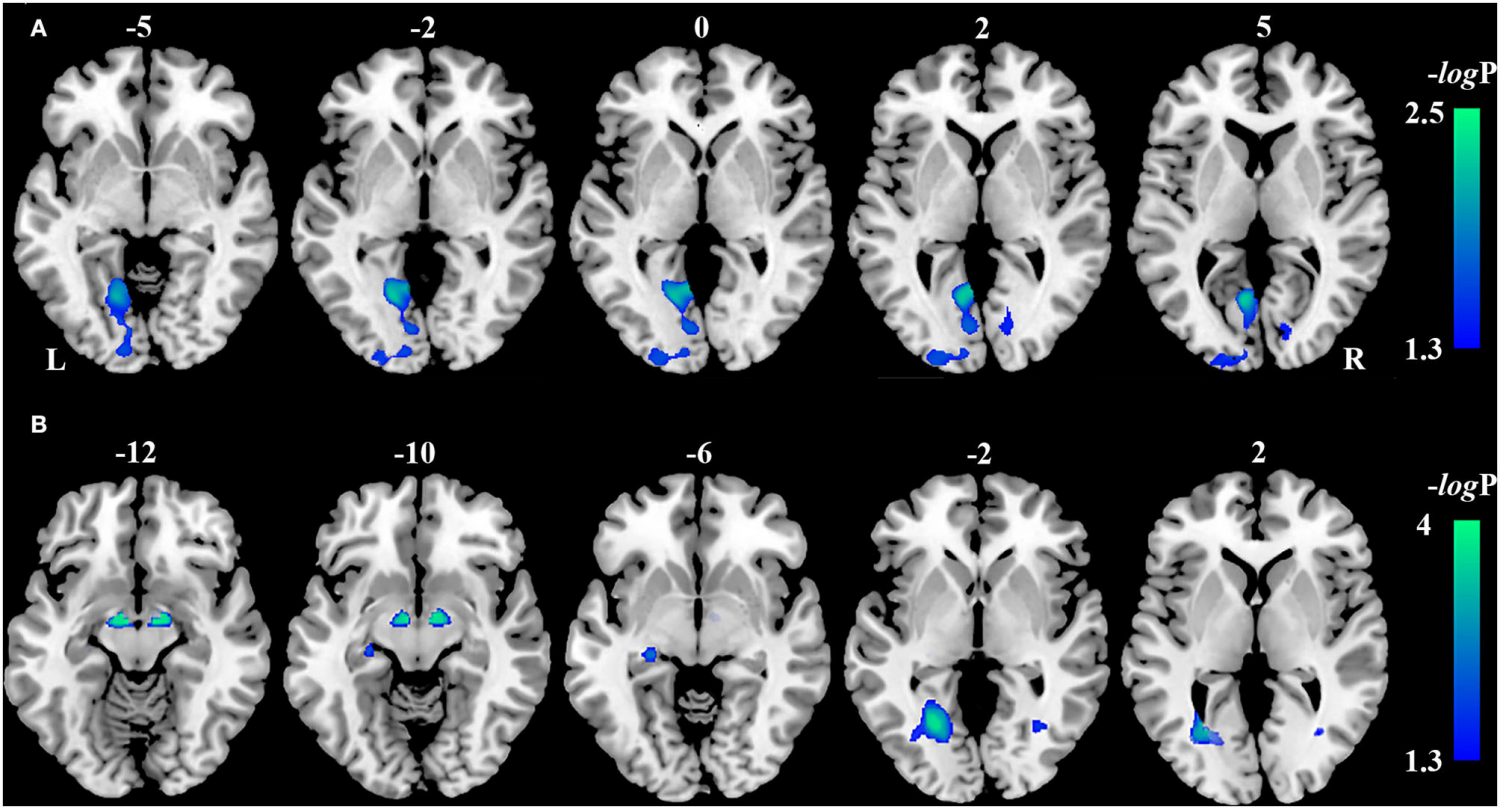
Inter-group differences in gray matter volume (GMV) and white matter volume. **(A)** The difference in GMV between the LHON and HC was tested using a voxel-wise non-parametric permutation test corrected for age and gender (*p* < 0.05, TFCE-FWE corrected). Chronic LHON had significantly lower GMV in the bilateral CG than in the HC (cool color). **(B)** The difference in WMV between the LHON and HC was tested using a voxel-wise non-parametric permutation test corrected for age and gender (*p* < 0.05, TFCE-FWE corrected). Chronic LHON had significantly lower WMV in the bilateral OR, bilateral OT, and left LGN than in the HC (cool color). Color bar represents the -log(p) value. CG, calcarine gyrus; FWE, family-wise error; GMV, gray matter volume; HC, healthy controls; LGN, lateral geniculate nucleus; LHON, Leber's hereditary optic neuropathy; OR, optic radiation; OT, optic tract; TFCE, threshold-free cluster enhancement; WMV, white matter volume.

**Figure 2:**
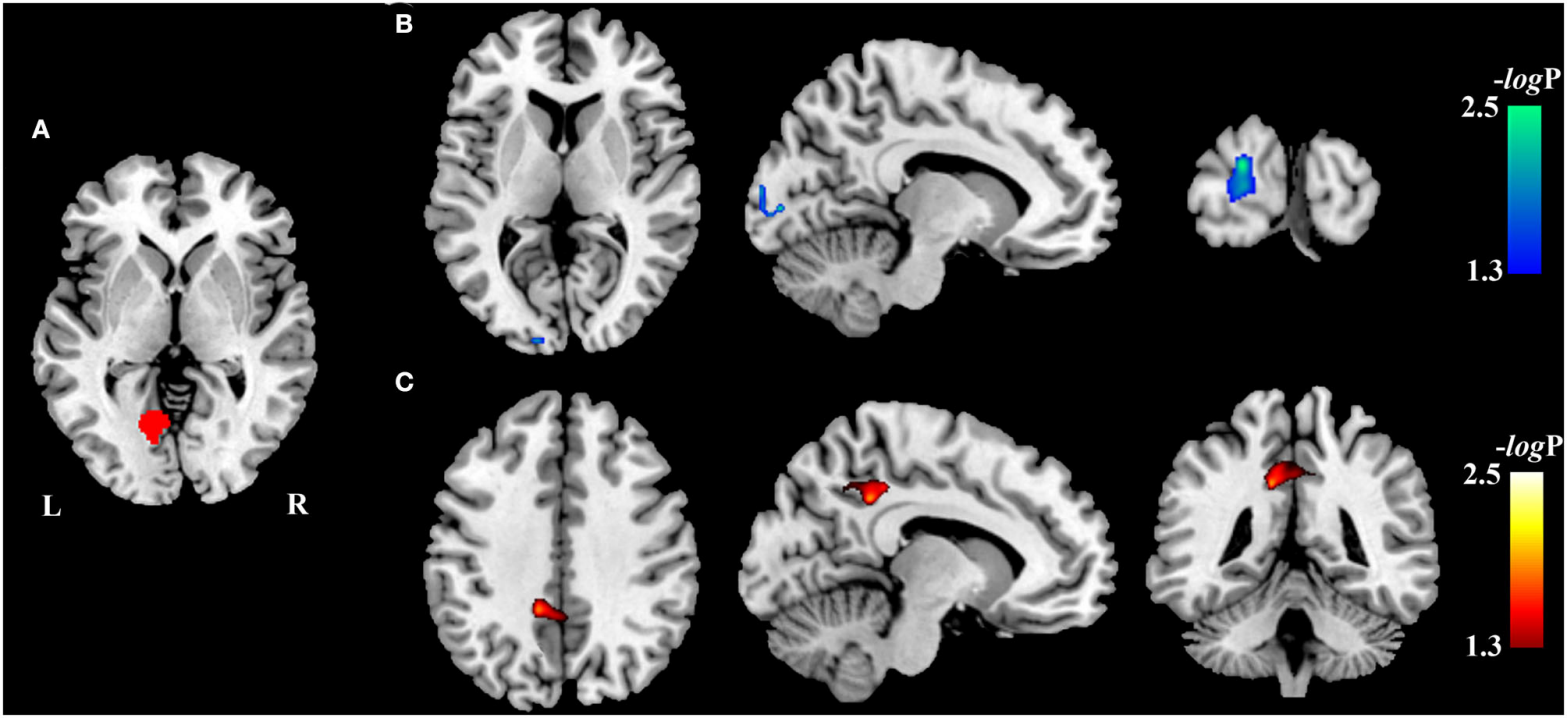
Inter-group differences in resting-state functional connectivity. The difference in FC between the chronic LHON and HC was test using a voxel-wise non-parametric permutation test corrected for age and gender (*p* < 0.05, TFCE-FWE corrected). Color bar represents the -log10(p) value. **(A)** Seeds (left CG) used to calculate the FC, **(B)** brain region (left SOG, cool color) having lower FC with the left CG in the chronic LHON, and **(C)** brain region (left PCC, warm color) having higher FC with the left CG. CG, calcarine gyrus; FC, functional connectivity; FWE, family-wise error; HC, healthy controls; LHON, Leber's hereditary optic neuropathy; PCC, posterior cingulate cortex; SOG, superior occipital gyrus; TFCE, threshold-free cluster enhancement.

Compared with the HCs, VBM analysis showed that chronic LHON had significantly lower GMV in the bilateral CG (non-parametric permutation test, *p* < 0.05, TFCE-FWE corrected) (Figure 1A, Table 2). In addition, VBM analysis showed that chronic LHON had significantly lower WMV in the bilateral optic tracts and optic radiations, and left LGN than those of the HCs (non-parametric permutation test, *p* < 0.05, TFCE-FWE corrected), as shown in Figure 1B, Table 2. We then defined the ROIs with changed GMV as seeds (left CG and right CG) and then calculated their FC at a voxel-wise level. Voxel-wise non-parametric permutation test identified that the LHON had significantly lower FC between the left CG and ipsilateral SOG and higher FC between left CG and the ipsilateral posterior cingulate gyrus (posterior cingulate cortex [PCC]) (*p* < 0.05, TFCE-FWE corrected) (Figure 2, Table 2).

**Table 2:** Intergroup differences in gray matter volume, white matter volume and functional connectivity.

**Metric**	**Brain region**	**Cluster size** **(ml)**	**Peak MNI coordinates**			***−log10*(p)**
			**x**	**y**	**z**	
GMV	Left CG	7.55	−10.5	−66	3	2.28
	Right CG	0.71	13.5	−79.5	0	1.48
WMV	Left OR	1.05	−28.5	−64.5	0	3.70
	Right OR	0.18	34.5	−66	0	2.09
	Left OT	0.20	−13.5	−9	−12	3.70
	Right OT	0.22	6	−9	−10.5	3.70
	Left LGN	0.18	−25.5	−25.5	−7.5	2.80
FC (left CG)	Left SOG	0.32	−12	−96	3	1.43
	Left PCC	1.76	−9	−42	39	1.80

*p values are corrected using TFCE-FWE method. FC, functional connectivity; CG, calcarine gyrus; FEW, family-wise error; GMV, gray matter volume; HC, healthy controls; LGN, lateral geniculate nucleus; LHON, Leber hereditary optic neuropathy; OR, optic radiation; OT, optic tract; PCC, posterior cingulate cortex; SOG, superior occipital gyrus; TFCE, threshold-free cluster enhancement; WMV, white matter volume*.

### Associations Between Neuroimaging Findings and Clinical Measures

**Figure 3:**
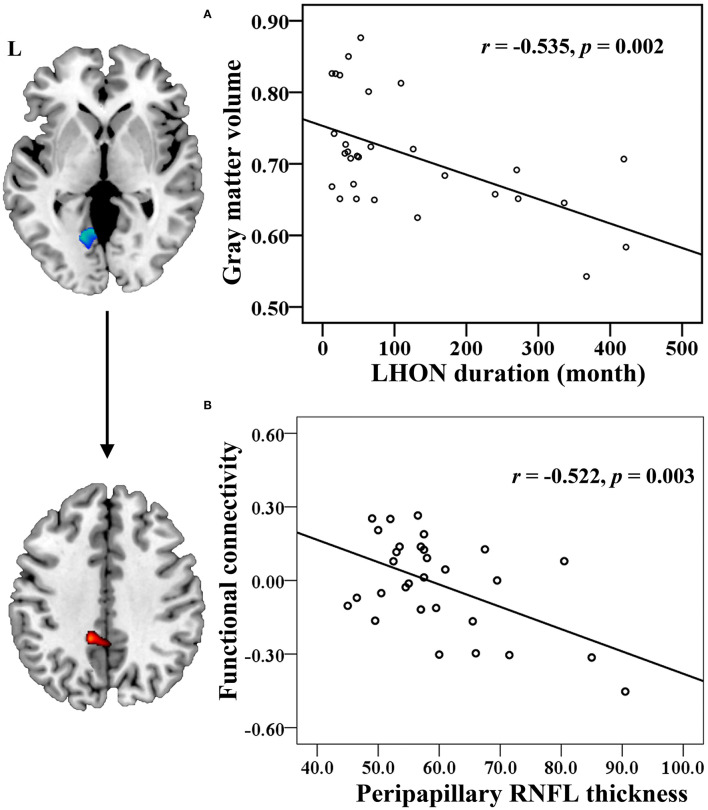
Correlation between the neuroimaging findings and clinical measures in the chronic LHON. **(A)** Spearman correlation coefficient was used to test the association between LHON duration and GMV of left CG, and **(B)** Pearson correlation coefficient was used to test that between peripapillary RNFL thickness and FC between the left CG and left PCC (*p* < 0.05, Bonferroni correction). CG, calcarine gyrus; FC, functional connectivity; GMV, gray matter volume; LHON, Leber's hereditary optic neuropathy; PCC, posterior cingulate cortex; RNFL, retinal nerve fiber layer.

The Spearman correlation showed that the GMV of the left CG was negatively correlated with the LHON duration (r = −0.535, *p* = 0.002, Bonferroni corrected) (Figure 3A). The Pearson correlation identified a negative correlation between the FC of left CG with ipsilateral PCC and the peripapillary RNFL thickness in the chronic LHON (r = −0.522, *p* = 0.003, Bonferroni corrected) (Figure 3B). There were no statistical differences between the remaining neuroimaging findings and clinical measures (*p* > 0.05).

## Discussion

In this study, we aimed to elucidate if the atrophy of the visual cortex in the chronic LHON would cause FC reorganization in the remote brain areas. We found that the GMV of bilateral CGs was significantly lower than the HCs. Taking these atrophied areas as seeds, we further found that the left CG had lower FC with the ipsilateral SOG and had higher FC with the ipsilateral PCC. Finally, besides the negative correlation between disease duration and GMV of left CG, we found a significantly negative correlation between the peripapillary RNFL thickness and the enhanced left CG-PCC FC in the chronic LHON. These findings indicate that the functional organization of the primary visual cortex has been reshaped in the chronic LHON.

Compared with HC subjects, we found that the patients with chronic LHON exhibited decreased GMV in bilateral CGs, which is consistent with the early studies on albinism (von dem Hagen et al., 2005), amblyopia (Mendola et al., 2005), LHON (Barcella et al., 2010), and retinal damage (Kitajima et al., 1997; Boucard et al., 2009). Similar to the LHON, reduced GMV of the visual cortex was also present in the blind people according to visual deprivation (Noppeney et al., 2005; Ptito et al., 2008; Qin et al., 2013; Yang et al., 2014; Jiang et al., 2015). Except for the results of reduced GMV of the primary visual cortex, previous neuroimaging studies have also reported decreased WMV and impaired WM integrity in the optic radiation in the blind and LHON individuals (Barcella et al., 2010; Milesi et al., 2012; Rizzo et al., 2012; Ogawa et al., 2014; Manners et al., 2015). Our study was also consistent with the previous findings, showing decreased fractional anisotropy of the optic radiations in the LHON (Wang et al., 2021). The reduced WMV of LGN and the atrophy of bilateral optic tracts were found in the patients with chronic LHON. In combination with the GM and WM atrophy of the primary visual cortex found in our results, the structural atrophy along the retinofugal pathway may be explained by downstream secondary transneuronal degeneration after the primary involvement of the RGCs in the chronic LHON.

The between-group comparison showed a lower FC between the left CG and the ipsilateral SOG, which might reflect a functional disconnection between the visual cortices in the visual network. As is known, LHON is characterized as RGC degeneration, finally leading to central vision loss (Carelli et al., 2004; Yu-Wai-Man et al., 2009; Giordano et al., 2011). The central vision loss was associated with functional damage to the visual cortex, which is likely secondary to RGC degeneration. Many previous studies have shown FC reduction between the visual cortical areas in patients with amblyopia (Mendola et al., 2018), diabetic retinopathy (Cheng et al., 2021), and retinal damage (Dai et al., 2013), although the coexistence of increased and decreased FC in the visual cortex was also reported (Rocca et al., 2011; Qin et al., 2013). In addition, several studies have reported the degeneration of the visual pathway and the visual cortex after visual deprivation, including a reduced fractional anisotropy in optic radiations (Wang et al., 2013), decreased FC between the visual cortices (Liu et al., 2007), and a decreased FC density in V1. In combination with the results of these previous studies, we speculated that the degenerative mechanisms may account for functional disconnectivity between the left CG and the ipsilateral SOG in the patients with chronic LHON.

In addition, the most interesting result of our findings was the left CG of the patients with chronic LHON had higher FC with the ipsilateral PCC than the sighted controls. The result was consistent with the previous findings, showing enhanced FC between the PCC and primary visual cortex in patients with type 2 diabetes mellitus (T2DM) (Cheng et al., 2021), increased FC density of the PCC in subjects with peripheral visual deprivation at the late age (Qin et al., 2015), and strengthened FC between the PCC and auditory cortex in the deafness (Malaia et al., 2014). Additional correlation analysis showed that the peripapillary RNFL thickness was negatively correlated to the enhanced FC between the left CG and PCC in the chronic LHON, indicating that more severe visual impairment would cause higher FC enhancement, in line with a prior study, showing patients with T2DM had a positive association between the PCC-calcarine FC and HbA1c, a blood indicator on the severity of T2DM (Cheng et al., 2021). Thus, these studies implied that long-term sensory impairment would remold the FC between the PCC and the deprived sensory area. As indicated by Kravitz et al., the dorsal visual stream can be separated into three major branches outside the occipital areas, including the visual-parieto–prefrontal pathway that mainly processes spatial working memory, the visual-parieto–premotor pathway that participants in visually guided action, and the visual-parieto–medial temporal pathway that is involved in spatial navigation (Kravitz et al., 2011). As a core hub of the visual-parieto–medial temporal pathway, the PCC has been indicated as an important relay between the visual cortex and hippocampus/medial temporal lobe to transport both bottom-up and top-down signals. An early study reported that blind people had increased FC between the posterior hippocampal subregions and PCC (Ma et al., 2016). In cooperation with the findings of enhanced FC between the PCC and deprived visual area, we speculated that top-down neural mechanism might account for functional remodeling of the FC between PCC and visual cortex in the chronic LHON through the dorsal visual stream. Further studies may be preferable to obtain direct evidence using the classical and specific cognitive tasks.

Based on the significant structural impairment of both the GM and WM of the visual cortex and strengthened FC between the PCC and the impaired visual area in patients with chronic LHON, recent studies have reported that the vision of the LHON patients can be restored through gene therapy (Sundaramurthy et al., 2021), and reversing blindness with gene therapy promotes long-term structural plasticity in the visual pathways of Leber's congenital amaurosis (Ashtari et al., 2015). The visual areas with atrophied GM or WM found in this study would be considered as potential targets to evaluate and monitor the therapeutic effects. However, chronic visual deprivation would cause secondary degeneration along the visual pathway, which may hinder the restoration of the visual function even when the frontier visual pathway (e.g., the RGC and optic nerves) is repaired. Thus, it is expected to introduce gene therapy as early as possible. Finally, as reported by early studies, cross-modal plasticity may be maladaptive for sight restoration. They reported persisting cross-modal changes in sight-recovery individuals with congenital cataracts (Guerreiro et al., 2016), and limited recovery of the visual function in sight-restored subjects (Fine et al., 2003; McKyton et al., 2015). The enhanced FC between the PCC and visual cortex in chronic LHON may be considered an indicator for the restoration of functional plasticity after therapy.

## Conclusion

In this study, we found that the GMV of bilateral CGs was atrophied in the chronic LHON. Moreover, the atrophied primary visual cortex was accompanied by functional dysconnectivity with ipsilateral SOG and strengthened connectivity with the default mode network. These findings indicate that the functional organization of the primary visual cortex has been reshaped in the chronic LHON.

## Data Availability Statement

The raw data supporting the conclusions of this article will be made available by the authors, without undue reservation.

## Ethics Statement

The studies involving human participants were reviewed and approved by the Ethics Committee of Henan Provincial People's Hospital. The patients/participants provided their written informed consent to participate in this study.

## Author Contributions

QT designed the study, collected the data, and drafted this article. LW and YZ analyzed the data and visualized result. KF collected the data and revised draft. ML analyzed the data. DS collected the data, supervised the project, and revised the draft. WQ designed the study and revised the draft. HD designed the study, analyzed the data, drafted, and revised this article. All authors contributed to the article and approved the submitted version.

## Funding

This study was supported by the Natural Science Foundation of China (81971599, 81771818, 81571659, 81271534, and 81601473), the Natural Science Foundation of Tianjin City (19JCYBJC25100 and 17JCYBJC29200), the Postdoctoral Research Foundation of China (2017M611175), and the Science & Technology Development Fund of Tianjin Education Commission for Higher Education (2020KJ207).

## Conflict of Interest

The authors declare that the research was conducted in the absence of any commercial or financial relationships that could be construed as a potential conflict of interest.

## Publisher's Note

All claims expressed in this article are solely those of the authors and do not necessarily represent those of their affiliated organizations, or those of the publisher, the editors and the reviewers. Any product that may be evaluated in this article, or claim that may be made by its manufacturer, is not guaranteed or endorsed by the publisher.
